# BMC reproductive health: family planning global conference series

**DOI:** 10.1186/s12978-016-0116-1

**Published:** 2016-02-06

**Authors:** Michael T Mbizvo, Anne Burke

**Affiliations:** 1 Population Council, Reproductive Health & Research, WHO/HQ, College of Health Science, University of Zimbabwe, Geneva, Switzerland; 2 Department of Population, Family, Reproductive Health, Johns Hopkins Bloomberg School of Public Health, Bayview Medical Center, 4940 Eastern Avenue, 21224 Baltimore, MD USA

## Editorial

Despite known benefits of modern contraception and family planning, especially in protecting the health of women and asserting their reproductive rights, more than 225 million women around the globe have an unmet need for wanting to avoid an unintended pregnancy [1]. Family planning could prevent up to 30 % of the more than 287,000 maternal deaths that take place globally every year [2], with low and middle income countries accounting for up to 99 % of the deaths.

The global conference series: International Family Planning Conference, first launched in Uganda in 2009, brought focus to research that is required to guide contraceptive method mix, improve quality of care, expand contraceptive access, family planning programming and coverage, as well as aid the development of new methods to meet the evolving needs of both new and current users. The subsequent 2012 London Family Planning Summit and its follow-on Family Planning 2020 initiative, further renewed impetus on family planning, with high-level policymaking buy-in. At this Summit, significant global and national commitments were made. These global developments require complementary scientific endeavour that can inform policy formulation and family planning program development or strengthening. A platform dedicated to sharing state of the art research ensures a scientific and policy environment that is responsive to critical elements for sustainable development. Implementation science research further ensures the identification of barriers to contraceptive method uptake and elaboration of factors associated with method switching or discontinuation.

Improving quality of care in family planning service delivery for example, through accessible expanded method-mix, as well as the training and wider distribution of providers, e.g. through skilled community distributors, as part of task shifting [3–5] are among key determinates of improving contraceptive prevalence rate (CPR). The premise that underpins increasing CPR is that improved quality of family planning services, e.g., through widening contraceptive choice, providing full information and counseling, based on plausible research findings, and client satisfaction with method, are among key determinants of contraceptive uptake and method use continuation. Full contraceptive access also requires provision of services to women across the reproductive lifespan, from adolescents to adulthood. The papers presented in this thematic series reflect a focus on some of these important considerations.

This series collates some of the papers presented during the 2013 International Family Planning Conference, which was attended by over 3,500 researchers, program implementers, policy makers, advocates, youth leaders and media and representatives of local and international organizations from 110 countries. The conference reflected on the theme “Full Access, Full Choice” to life saving Family Planning information, supplies and service. The papers presented all have the objective of responding to the contraceptive needs of the millions of women which in some instances, is a matter of survival or death.

These responses can take a variety of approaches, including harnessing mobile health technology to support family planning access in Cambodia (Smith et al. [6]), and assessing feasibility of contraceptive service integration into the workload of community health workers in Rwanda (Chin Quee et al. [7]). Understanding women’s fertility desires and contraceptive needs, as explored among women in 3 peri-urban communities in sub-Saharan Africa (OlaOlorun et al. [8]), is an important component in the evolution of successful family planning programs. And we must understand the contraceptive needs of women at all stages of reproductive life, which reflect a growing recognition of the importance of youth- and adolescent-friendly reproductive health services as espoused in an analysis of data from 8 low- and middle-income countries in the paper by Chandra-Mouli et al. [9]. These studies are part of the global to effort to advance sexual and reproductive health through, among other things, appropriately addressing the wider contraceptive needs of women and couples throughout the reproductive life span.
